# The Association Between Disgust Sensitivity and Negative Attitudes Toward Homosexuality: The Mediating Role of Moral Foundations

**DOI:** 10.3389/fpsyg.2019.01229

**Published:** 2019-06-06

**Authors:** Ruile Wang, Qi Yang, Peng Huang, Liyang Sai, Yue Gong

**Affiliations:** ^1^Department of General Education, Xiamen University Tan Kah Kee College, Zhangzhou, China; ^2^School of Humanities, Tongji University, Shanghai, China; ^3^Department of Military Medical Psychology, Air Force Medical University, Xi’an, China; ^4^Department of Psychology, Hangzhou Normal University, Hangzhou, China; ^5^School of Psychology, Shaanxi Normal University, Xi’an, China

**Keywords:** disgust sensitivity, attitudes toward homosexuality, moral foundations, sanctity, authority

## Abstract

Previous studies have found that “disgust-sensitive” individuals have negative attitudes toward gay and lesbian people, but the underlying mechanisms for such attitudes remain unclear. Based on moral foundations theory, the current paper assumes that the relationship between disgust sensitivity and attitudes toward homosexuality are mediated by moral foundations. In order to test this assumption, the current authors examined the questionnaire answers from a total of 452 Chinese undergraduates who participated in this study. The results showed that disgust sensitivity was positively correlated with negative attitudes toward homosexuality, and positively correlated with moral concerns in five domains (care, fairness, loyalty, authority, and sanctity). Authority and sanctity were both associated with attitudes toward homosexuality, while only sanctity mediated the relationship between disgust sensitivity and attitudes toward homosexuality. Overall, the results suggest that considering moral foundations (especially sanctity) may lend more insight to the associations between disgust sensitivity and negative attitudes toward gay and lesbian people.

## Introduction

Over the past few years, several countries, such as the United States and Finland, have begun to legally recognize same-sex marriages. Alongside this, the general opinion of gay and lesbian people seems to have improved. A public survey in 39 countries, conducted by the Pew Research Center (2013), found that homosexuality was broadly accepted in North America, the European Union, and much of Latin America. Nevertheless, prejudice toward homosexuals is still a common social problem in many countries. The Pew Research Center (2013) also found a broad widespread rejection of homosexuality in Russia, Africa, some parts of Asia, and predominantly Muslim nations. In China, majorities (57%) reported the negative attitude that homosexuality should not be accepted by the society, while just 21% believed homosexuality should be accepted. Public attitudes toward homosexuality (i.e., attitudes toward lesbians and gay men, or ATLG, for its acronym) affect social policies on homosexuality and gay and lesbian rights (Lin et al., 2016). In 2014, several state legislatures in United States passed controversial laws that allowed the refusal of business service to gay and lesbian people based on religious objections (Sanchez and Marquez, 2014). Thus, exploring how and to what extent certain factors influence attitudes toward homosexuality is vital in enabling more effective policy making and collective action to improve worldwide awareness of gay rights.

“Disgust” is an emotion induced by repulsive stimuli. It may be evoked through different sensory routes, including taste, smell and vision (Terrizzi et al., 2010). Numerous studies have examined the relationship between disgust and prejudice toward homosexuals, showing that a dispositional proneness to disgust (“disgust sensitivity” or “trait disgust”; Haidt et al., 1994) is associated with negative attitudes toward homosexuality (e.g., Inbar et al., 2009b; Terrizzi et al., 2010; Crawford et al., 2014). A meta-analysis of 17 studies examining the association between disgust and prejudice toward gay men (i.e., homonegativity) revealed that the average effect size found in studies examining the relationship between disgust sensitivity and homonegativity was moderate (*d* = 0.64), and moderate to large (*d* = 0.77) in studies investigating the relationship between induced disgust and homonegativity (Kiss et al., 2018). People who are more prone to experiencing disgust are more likely to be politically and socially conservative (Inbar et al., 2009a, 2012b), and thus show more negative attitudes toward homosexuals (Inbar et al., 2009b; Terrizzi et al., 2010; Crawford et al., 2014). However, although a link between disgust sensitivity and prejudice toward homosexuality has been found to exist, the underlying mechanism and processes which establish it remain vague. One possibility has to do with the finding that individuals who are more disgust-sensitive generally hold more conservative views about sex (Olatunji, 2008). Crawford et al. (2014) provided solid support for this finding. They discovered that disgust sensitivity to contamination affected the adoption of negative attitudes toward groups perceived to threaten traditional sex-related morality (e.g., abortionist), and positive attitudes toward those upholding such morality (e.g., young people who maintain celibacy until marriage). Crawford et al. (2014) suggested that prejudice against the gay and lesbian people is just part of a broader set of negative attitudes toward groups seen as threatening to traditional sex-related morality. The present research investigated whether the relationship between disgust sensitivity and prejudice toward homosexuality is explained in part by moral values.

Moral psychology distinguishes between moral content and moral judgment, defining what morality is and how it works (Cameron et al., 2015). Individuals might condemn a person who hurt others or jumped a queue as immoral. However, these moral judgments involve different moral content, with hurting others involving “care” and jumping the queue involving “justice” (Graham et al., 2013). The moral foundations theory proposes that human groups construct moral virtues, narratives and institutions based on five (or more) innate and universally available psychological systems (or “foundations”): care, fairness, loyalty, authority and sanctity (the latter also being termed purity), with liberty as a potential sixth system (Haidt, 2012; Graham et al., 2013). For instance, the care system, which was shaped by the psychology of childcare, underlies the virtues of caring and kindness, and explains why we intuitively judge hurting a child to be immoral. The foundation of care evokes feelings of compassion for victims and anger toward those who hurt others. The system of sanctity was shaped by the psychology of communicable diseases avoidance, which underlies the virtues of temperance, chastity, piety, and explains why some people intuitively feel that sibling incest is morally wrong and disgusting (Graham et al., 2013). Similarly, we condemn cheating (fairness system), disloyalty to members of an in-group (loyalty system), or disrespect toward authorities (authority system). Each moral foundation produces “fast gut feelings” of like and dislike rather than thoughtful deliberation (Haidt, 2001).

Based on the moral foundations theory, the focus of the current paper is more on the foundation of sanctity than the other four moral foundations. From an evolutionary perspective, contemporary researchers generally agree that disgust is a protective mechanism that helps individuals to avoid a toxic or infectious situation, object or person, which is an important component of the “behavioral immune system” (Murray and Schaller, 2016). According to the moral foundations theory, the emotion of disgust and the sanctity foundation were both evolved to avoid pathogens (Haidt, 2012). The behavioral immune system are marked by a tendency to overgeneralize. That is, they respond to an overly general set of superficial cues, which can result in aversive responses to things (including people) that pose no actual threat of pathogen infection (Murray and Schaller, 2016; Ackerman et al., 2018). Therefore, it is reasonable to hypothesize that the foundation of sanctity may partially explained the relationship between disgust sensitivity and attitudes toward homosexuality. That is, those people prone to experiencing disgust may be more concerned with the foundation of sanctity, and thus see homosexuality as nasty, impure and immoral.

Previous studies have found that the associations between disgust sensitivity, moral foundations and prejudice toward homosexuality are robust. Disgust has consistently been related to moral values (Van Leeuwen et al., 2017). However, it remains unclear how individual differences in disgust sensitivity affects moral judgment across different moral domains. One perspective suggests that disgust sensitivity is specifically linked to sanctity. This “specific” perspective is consistent with the moral foundations theory, which proposes a specific emotion for each moral foundation (Graham et al., 2013). Specifically, sanctity-based transgressions elicit feelings of disgust. The “specific” perspective has been examined by some empirical studies (Horberg et al., 2009; Inbar et al., 2009a; Wagemans et al., 2018). For example, Wagemans et al. (2018) found that disgust sensitivity was more closely related to moral concerns of sanctity than to moral concerns of any other domain (i.e., care, fairness, loyalty, authority, and liberty). Other perspectives imply a more general relationship between disgust sensitivity and moral foundations, indicating that disgust sensitivity may be associated with multiple moral foundations (Chapman and Anderson, 2013; Tybur et al., 2013; Cameron et al., 2015). Some data are consistent with this “general” perspective. For example, Chapman and Anderson (2014) discovered that disgust sensitivity was positively correlated with the moral condemnation of care-based transgressions and fairness-based transgressions. A recent study found that each type of disgust sensitivity was uniquely associated with at least one moral domain: moral disgust was associated with all five moral foundations (the largest predictive effect exerted on fairness); sexual disgust was associated with care, loyalty, authority, and sanctity (the largest predictive effect exerted on sanctity); while pathogen disgust had small predictive effects for loyalty, authority, and sanctity (Van Leeuwen et al., 2017).

Some evidence suggests that moral foundations are also linked to attitudes toward homosexuality (Koleva et al., 2012; Barnett et al., 2018). Haidt and Graham (2007) argued that conservative opposition to same-sex marriage stems from the moral concerns of binding foundations (i.e., foundations that emphasize morality in the context of a group or collective, such as loyalty, authority and sanctity), whereas liberal support stems from concerns pertaining to individualizing foundations (i.e., foundations that emphasize the rights of the individual over broader group-related interests, such as care and fairness). Koleva et al. (2012) tested this assertion empirically and found that *sanctity* emerged as the strongest predicting foundation for a variety of issues, such as casual sex, pornography, same-sex relationships/marriage, and births out of wedlock. However, Barnett et al. (2018) presented a different picture, in which negative attitudes toward homosexuality were positively correlated with an endorsement of binding foundations, and negatively correlated with an endorsement of individualizing foundations.

As reviewed above, to date, most of the studies on the relationship between disgust, morality and ATLG have been conducted in so-called “WEIRD” cultures (Western, Educated, Industrialized, Rich, and Democratic; Graham et al., 2013). The current study used a less WEIRD sample from China in order to examine whether patterns observed in WEIRD populations generalize to other populations. Furthermore, given that significant relationships have been uncovered between disgust sensitivity and attitudes toward homosexuality, and that moral foundations have been found to be closely linked to disgust sensitivity and attitudes toward homosexuality, it is reasonable to hypothesize that moral foundations (especially sanctity) may mediate the relationship between disgust sensitivity and attitudes toward homosexuality. Generally speaking, this study aims to investigate the association between disgust sensitivity and negative attitudes toward homosexuality, as well as the mediating role of moral foundations (especially sanctity) in terms of this link.

## Materials and Methods

### Participants

A total of 492 participants were invited from two Chinese universities. The final valid sample in this study comprised 452 students (355 females, 97 males; *M_age_* = 19.70 years, *SD* = 1.34 years). 40 students were excluded because 27 of them did not fill out the questionnaires carefully according to the polygraph items, and 13 declared themselves to be non-heterosexual. Informed consent was implied through survey completion.

### Measures

The Chinese version of the 30-item Moral Foundations Questionnaire (MFQ30^1^) was used to measure the endorsement of moral foundations. The MFQ30 consists of two sections. In the first section, the participants were asked to rate how relevant 16 various considerations are when they make decisions about whether something is morally right or wrong (e.g., “Whether or not someone cared for someone weak or vulnerable”), on a 6-point scale, ranging from 0 (“not at all relevant”) to 5 (“extremely relevant”). In the second section, the participants rated their agreement or disagreement with 16 statements (e.g., “People should not do things that are disgusting, even if no one is harmed”), on a 6-point scale, ranging from 0 (“strongly disagree”) to 5 (“strongly agree”). The MFQ30 yields one subscale for each of the five moral foundations. Higher scores indicate a higher endorsement of that particular moral foundation. The Cronbach’s alphas for the five subscales were found to be acceptable in this study: Care (α = 0.43), Fairness (α = 0.64), Loyalty (α = 0.60), Authority (α = 0.55), Sanctity (α = 0.51). The alphas were not as high as in many other scales, especially for the Care subscale. Improving the reliability would have required dropping some items; however, all items were retained, as recommended by Graham et al. (2011), because the goal of the MFQ30 is to measure an expansive range of moral concerns with a small number of items across two different item formats.

The 20-item Attitudes toward Lesbians and Gay Men Scale (ATLG; Herek, 1998; modified for the Chinese context by Yu et al., 2010) was used to measure attitudes toward homosexuality. This scale consists of 20 different statements – 10 items about gay men (ATG) and 10 about lesbians (ATL). Each item is rated on a 5-point scale, ranging from 0 (“strongly disagree”) to 4 (“strongly agree”). Higher scores indicate more negative attitudes. In this study, the Cronbach’s alphas for ATG, ATL and ATLG were 0.92, 0.94, and 0.96, respectively.

The 25-item Disgust Scale-Revised (DS-R; Haidt et al., 1994; modified by Olatunji et al., 2007), was used to measure disgust sensitivity. This scale contains three dimensions of disgust: core disgust, animal reminder disgust, and contamination-based disgust. This scale was deemed to be a more suitable choice for the present study than other scales measuring disgust sensitivity because the DS-R did not include items concerning sexual disgust. Translation and back translation procedures were used to ensure that the Chinese translation represented an accurate version of the English version of the DS-R. The participants were asked to rate their agreement or disagreement with 13 statements, and then to rate how disgusted the participants would be in 12 particular situations. Each item is rated on a 5-point scale, ranging from 0 (“strongly disagree” or “not at all disgusting”) to 4 (“strongly agree” or “very disgusting”). Higher scores indicate stronger disgust sensitivity. The Cronbach’s alpha coefficient for DS-R was 0.86 in the current study, and for core disgust, animal reminder disgust and contamination-based disgust was 0.74, 0.78, and 0.51, respectively. A confirmatory factor analysis showed an acceptable fit for the three-factor measurement model: χ^2^(272) = 799.12, GFI = 0.86, CFI = 0.79, RMSEA = 0.07, and the single-factor model that combined the three subdomains of disgust into one factor: χ^2^(277) = 961.17, GFI = 0.83, CFI = 0.73, RMSEA = 0.07. The bi-factor model, in which the items were set to load on their designated factors and also to cross-load onto an overall “disgust sensitivity” factor, also had an adequate fit: χ^2^(247) = 584.62, GFI = 0.90, CFI = 0.87, RMSEA = 0.06. The results showed that the bi-factor model had a superior fit to the single-factor and three-factor models.

### Procedure

The questionnaires were administered via an online survey. The participants were notified that all of their responses would only be accessible to the research group, after which they were asked to indicate their age, gender, and sexual orientation (heterosexual, homosexual, and bisexual), and to complete the MFQ30, the ATLG and the DS-R in turn. In order to eliminate social desirability bias, the participants were all anonymous in the online survey. All procedures were approved by the board of ethics at the Department of General Education, Xiamen University Tan Kah Kee College.

### Data Analysis

This was a cross-sectional survey study. Descriptive statistics and correlation analysis were calculated using SPSS 21.0. Hayes (2013) PROCESS for SPSS, a bootstrapping procedure, was used to test and calculate the mediating effect, with *N* = 10000 bootstrap samples. The mediation effects were statistically significant (*p* < 0.05) when the 95% confidence intervals (95% CI) did not include 0 (Hayes, 2013) (Supplementary Material).

### Common Method Bias

Common method bias (CMB) is a potential problem in behavioral research. This bias can be controlled through both procedural and statistical techniques (Podsakoff et al., 2003). Procedural remedies were addressed by protecting respondent anonymity, reducing evaluation apprehension, using reverse scored items and polygraph items. Harman’s one-factor test was conducted to test the hypotheses that a single factor emerges from the exploratory factor analysis or one general factor account for the majority of the covariance among the measures. The statistic test result showed that 18 factors in the unrotated factor structure with the first factor only accounting for 17.36% of the total variance explained. In summary, there was not significant CMB in the measurement.

## Results

### Correlation Analyses

Men were found to be less disgust sensitive than women [*M*_men_ = 57.55, *M*_women_ = 64.89, *t* (450) = 4.83, *p* < 0.001], for which reason the following analyses include gender as a covariate. The mean and standard deviation of each variable and partial correlations for all variables of interest are shown in Table 1. After controlling for gender (dummy coded, where 0 = female and 1 = male), disgust sensitivity was seen to be significantly positively correlated with each of the five moral foundations, and most strongly correlated with the foundation of sanctity. Disgust sensitivity was also positively correlated with ATG and ATL, with a stronger relationship on ATG. Sanctity was significantly correlated with attitudes toward homosexuality, as was expected. However, contrary to expectations, the correlation between authority and attitudes toward homosexuality also emerged as significant.

**Table 1:** Descriptive statistics and partial correlations among variables.

Variables	1	2	3	4	5	6	7	8	9	10	11	12
1 DS	—											
2 Core disgust	0.91^∗∗∗^	—										
3 Animal reminder disgust	0.82^∗∗∗^	0.58^∗∗∗^	—									
4 Contamination disgust	0.75^∗∗∗^	0.60^∗∗∗^	0.43^∗∗∗^	—								
5 Care	0.19^∗∗∗^	0.18^∗∗∗^	0.17^∗∗∗^	0.10^∗^	—							
6 Fairness	0.14^∗∗^	0.15^∗∗^	0.09^∗^	0.09^∗^	0.67^∗∗∗^	—						
7 Loyalty	0.11^∗^	0.14^∗∗^	0.08	0.03	0.61^∗∗∗^	0.61^∗∗∗^	—					
8 Authority	0.14^∗∗^	0.13^∗∗^	0.12^∗^	0.08	0.47^∗∗∗^	0.44^∗∗∗^	0.64^∗∗∗^	—				
9 Sanctity	0.26^∗∗∗^	0.22^∗∗∗^	0.24^∗∗∗^	0.18^∗∗∗^	0.48^∗∗∗^	0.47^∗∗∗^	0.54^∗∗∗^	0.58^∗∗∗^	—			
10 ATG	0.28^∗∗∗^	0.23^∗∗∗^	0.24^∗∗∗^	0.24^∗∗∗^	−0.02	−0.02	0.07	0.17^∗∗∗^	0.39^∗∗∗^	—		
11 ATL	0.19^∗∗∗^	0.13^∗∗^	0.21^∗∗∗^	0.17^∗∗∗^	−0.02	−0.02	0.08	0.16^∗∗^	0.37^∗∗∗^	0.81^∗∗∗^	—	
12 ATLG	0.25^∗∗∗^	0.19^∗∗∗^	0.24^∗∗∗^	0.22^∗∗∗^	−0.02	−0.03	0.08	0.17^∗∗∗^	0.40^∗∗∗^	0.95^∗∗∗^	0.95^∗∗∗^	—
Absolute range	25–125	12–48	0–32	0–20	0–30	0–30	0–30	0–30	0–30	0–40	0–40	0–80
M	63.31	29.92	23.48	9.91	18.07	17.54	18.10	15.99	16.15	15.41	12.79	28.20
SD	13.60	6.97	5.50	3.58	3.91	4.32	4.36	4.32	4.29	8.46	8.03	15.62

*DS, disgust sensitivity; ATG, attitudes toward gay men; ATL, attitudes toward lesbians; ATLG, Attitudes toward Lesbians and Gay Men. ^∗^p < 0.05; ^∗∗^p < 0.01; ^∗∗∗^p < 0.001.*

### Mediation Analyses

As shown in the correlation analyses above, in addition to sanctity, authority was also correlated with disgust sensitivity and attitudes toward homosexuality. For this reason, the mediation role of both authority and sanctity were tested.

In a multiple mediation model (model A), disgust sensitivity was the independent variable, authority and sanctity were the mediators, ATLG was the dependent variable, and gender was a covariate. The results showed that disgust sensitivity had a positive total association with ATLG [β = 0.29, *SE* = 0.06, *t* = 5.32, *p* < 0.001, 95% CI (0.18, 0.40)]. Disgust sensitivity had positive associations with authority [β = 0.04, *SE* = 0.02, *t* = 2.86, *p* < 0.01, 95% CI (0.01, 0.08)] and sanctity [β = 0.08, *SE* = 0.01, *t* = 5.79, *p* < 0.001, 95% CI (0.05, 0.11)]. Authority did not have a significant association with ATLG [β = −0.30, *SE* = 0.19, *t* = −1.60, *p* = 0.11, 95% CI (−0.67, 0.07)]. Sanctity had a positive association with ATLG [β = 1.50, *SE* = 0.21, *t* = 7.08, *p* < 0.001, 95% CI (1.08, 1.91)]. Together, disgust sensitivity, sanctity and authority explained 19.0% of the variance in ATLG. Disgust sensitivity had a positive direct association with ATLG [*β* = 0.18, *SE* = 0.05, *t* = 3.54, *p* < 0.001, 95% CI (0.08, 0.28)], and an indirect association with ATLG through sanctity [β = 0.12, *SE* = 0.03, 95% CI (0.07, 0.18)], but not through authority [β = −0.01, *SE* = 0.01, 95% CI (−0.04, 0.00), in that the 95% confidence intervals included 0]. The mediating path through sanctity accounted for 42.2% of the total association of disgust sensitivity with ATLG. Model A is illustrated in Table 2.

**Table 2:** Individual associations between variables in the mediation model where authority and sanctity were hypothesized to mediate the association between disgust sensitivity and ATLG (*N* = 452).

Predictors	Authority			Sanctity			ATLG		
	β	*t*	95%CI	β	*t*	95%CI	β	*t*	95%CI
Gender	0.60	1.06	[−0.51, 1.70]	0.05	0.10	[−0.97, 1.08]	3.75^∗^	2.32	[0.58, 6.92]
DS	0.04^∗∗^	2.86	[0.01, 0.08]	0.08^∗∗∗^	5.79	[0.05, 0.11]	0.18^∗∗∗^	3.54	[0.08, 0.28]
Authority							−0.30	−1.60	[−0.67, 0.07]
Sanctity							1.50^∗∗∗^	7.08	[1.08, 1.91]
*R*^2^		0.02^∗^			0.07^∗∗∗^			0.19^∗∗∗^	
*F*		4.14			18.11			22.13	

*Each column set is a regression equation that predicts the criterion at the top of the column. ^∗^p < 0.05; ^∗∗^p < 0.01; ^∗∗∗^p < 0.001.*

Some previous studies found that disgust affected ALG but not ATL (e.g., Inbar et al., 2012a). In order to figure out whether the link between disgust sensitivity and ATL is mediated by moral foundations in the same way as the link between disgust sensitivity and ATG, two multiple mediation models (model B and model C) were run. These were identical to model A, except that ATG and ATL were the respective dependent variables. As with model A, disgust sensitivity was found to have positive total associations with authority and sanctity in models B and C. The results of model B showed that disgust sensitivity had a positive total association with ATG [β = 0.18, *SE* = 0.03, *t* = 6.02, *p* < 0.001, 95% CI (0.12, 0.23)]. Authority did not have a significant association with ATG [β = −0.15, *SE* = 0.10, *t* = −1.48, *p* = 0.14, 95% CI (−0.36, 0.05)]. Sanctity had a positive association with ATG [β = 0.77, *SE* = 0.11, *t* = 7.30, *p* < 0.001, 95% CI (0.56, 0.97)]. Disgust sensitivity had a positive direct association with ATG [β = 0.12, *SE* = 0.03, *t* = 4.37, *p* < 0.001, 95% CI (0.07, 0.17)], and an indirect association with ATG through sanctity [β = 0.06, *SE* = 0.01, 95% CI (0.04, 0.09)], but not through authority. The mediating path through sanctity accounted for 35.8% of the total association of disgust sensitivity with ATG.

The results of model C showed that disgust sensitivity had a positive total association with ATL [β = 0.12, *SE* = 0.03, *t* = 4.04, *p* < 0.001, 95% CI (0.06, 0.17)]. Authority did not have a significant association with ATL [β = −0.15, *SE* = 0.10, *t* = −1.44, *p* = 0.15, 95% CI (−0.35, 0.05)]. Sanctity had a positive association with ATL [β = 0.73, *SE* = 0.12, *t* = 6.17, *p* < 0.001, 95% CI (0.50, 0.96)]. Disgust sensitivity had a positive direct association with ATL [β = 0.06, *SE* = 0.03, *t* = 2.26, *p* < 0.05, 95% CI (0.01, 0.12)], and an indirect association with ATL through sanctity [β = 0.06, *SE* = 0.01, 95% CI (0.03, 0.09)], but not through authority. The mediating path through sanctity accounted for 51.8% of the total association of disgust sensitivity with ATL. Models B and C are illustrated in Table 3.

**Table 3:** Individual associations between variables in the mediation model, where authority and sanctity were hypothesized to mediate the association between disgust sensitivity and ATG/ATL (*N* = 452).

Predictors	ATG			ATL		
	β	*t*	95%CI	β	*t*	95%CI
Gender	3.83^∗∗∗^	4.36	[2.10, 5.56]	−0.08	−0.09	[−1.83, 1.66]
DS	0.12^∗∗∗^	4.37	[0.07, 0.17]	0.06^∗^	2.26	[0.01, 0.12]
Authority	−0.15	−1.48	[−0.36, 0.05]	−0.15	−1.44	[−0.35, 0.05]
Sanctity	0.77^∗∗∗^	7.30	[0.56, 0.97]	0.73^∗∗∗^	6.17	[0.50, 0.96]
*R*^2^		0.21^∗∗∗^			0.15^∗∗∗^	
*F*		27.92			16.35	

*Each column set is a regression equation that predicts the criterion at the top of the column. ^∗^p < 0.05; ^∗∗∗^p < 0.001.*

In order to test the mediating role of sanctity on the relationship between the three domains of disgust sensitivity (i.e., core disgust, animal reminder disgust and contamination-based disgust) and ATLG, three mediation models were run, with the three domains of disgust as the independent variable. The results were similar regardless of which subdomain of disgust was used (see Tables 4–6).

**Table 4:** Individual associations between variables in the mediation model, where sanctity was hypothesized to mediate the association between core disgust and ATLG (*N* = 452).

Predictors	Sanctity			ATLG		
	β	*t*	95%CI	β	*t*	95%CI
Gender	−0.13	−0.26	[−1.16, 0.89]	2.99	1.85	[−0.19, 6.17]
Core disgust	0.14^∗∗∗^	4.75	[0.08, 0.19]	0.24^∗^	2.30	[0.03, 0.44]
Sanctity				1.38^∗∗∗^	8.17	[1.05, 1.72]
*R*^2^		0.05^∗∗∗^			0.17^∗∗∗^	
*F*		12.12			25.87	

*Each column set is a regression equation that predicts the criterion at the top of the column. ^∗^p < 0.05; ^∗∗∗^p < 0.001.*

**Table 5:** Individual associations between variables in the mediation model, where sanctity was hypothesized to mediate the association between animal reminder disgust and ATLG (*N* = 452).

Predictors	Sanctity			ATLG		
	β	*t*	95%CI	β	*t*	95%CI
Gender	0.07	0.14	[−0.95, 1.10]	3.65^∗^	2.26	[0.47, 6.83]
Animal reminder disgust	0.19^∗∗∗^	5.18	[0.12, 0.26]	0.43^∗∗∗^	3.53	[0.19, 0.67]
Sanctity				1.34^∗∗∗^	7.83	[1.00, 1.67]
*R*^2^		0.06^∗∗∗^			0.18^∗∗∗^	
*F*		14.50			30.29	

*Each column set is a regression equation that predicts the criterion at the top of the column. ^∗^p < 0.05; ^∗∗∗^p < 0.001.*

**Table 6:** Individual associations between variables in the mediation model, where sanctity was hypothesized to mediate the association between contamination-based disgust and ATLG (*N* = 452).

Predictors	Sanctity			ATLG		
	β	*t*	95%CI	β	*t*	95%CI
Gender	−0.34	−0.66	[−1.36, 0.67]	2.91	1.80	[−0.26, 6.08]
Contamination-based disgust	0.21^∗∗∗^	3.68	[0.10, 0.32]	0.66^∗∗∗^	3.33	[0.27, 1.05]
Sanctity				1.37^∗∗∗^	8.12	[1.04, 1.70]
*R*^2^		0.03^∗∗∗^			0.18^∗∗∗^	
*F*		7.72			29.11	

*Each column set is a regression equation that predicts the criterion at the top of the column. ^∗∗∗^p < 0.001.*

## Discussion

This study examines the association between disgust sensitivity and attitudes toward homosexuality, together with the mediating role of moral foundations on the link between disgust sensitivity and attitudes toward homosexuality. Consistent with prior research (Inbar et al., 2009b; Terrizzi et al., 2010), the results show that disgust sensitivity was positively related to negative attitudes toward homosexuality. In other words, individuals who are more sensitive to disgusting stimuli were found to be more negatively prejudiced toward the gay and lesbian population. More importantly, it emerged that disgust sensitivity had an indirect association with ATLG through sanctity, but not through authority or any other moral foundation. The results of models B and C suggest that sanctity mediates the relationship between disgust sensitivity and both ATG and ATL. Authority was not seen to mediate the relationship between disgust sensitivity and ATLG since this foundation did not have a significant association with ATLG.

In line with moral foundations theory, the current findings suggest that the relationship between disgust sensitivity and attitudes toward homosexuality is partially explained by moral concerns of sanctity. Taking an evolutionary approach, the moral foundations theory proposes that each moral foundation was shaped by a recurring adaptive challenge that human ancestors confronted over the course of our long evolutionary history (Graham et al., 2013). Specifically, the evolutionary origins of the foundation of sanctity might be the powerful selection pressures exerted by pathogens and parasites (Graham et al., 2013). To minimize the risk of infection, humans thus evolved not only a physiological immune system, but also a behavioral immune system, of which the emotion of disgust is the affective component. Based on these perspectives, the covariation between pathogen disgust sensitivity and the endorsement of sanctity may reflect a similar behavioral strategy that minimizes pathogenic infection risk by avoiding outgroups and consolidating ingroup cohesion. Disgust-sensitive individuals may be more likely to endorse the virtues of purity, in such a way that induces them to regard homosexuality as immoral and to produce an emotional reaction of disgust.

According to moral foundations theory, morality is native and sensitive to culture. That is, there exists a universal first draft of the moral mind, which draft is then revised in variable ways across cultures (Graham et al., 2013). In China, filial piety has been an essential moral value for thousands of years, originating from Confucianism (Lin et al., 2016). Of the five moral foundations, filial piety is clearly most related to the foundation of authority. Using international MFQ data, Graham et al. (2011) found that, compared with Western participants, participants from Eastern cultures (including the Chinese culture) showed stronger concerns about binding foundations that emphasize morality in the context of a group or collective (i.e., loyalty, authority, and sanctity).

One of the key strengths of this study is that its sample is drawn from Chinese undergraduates, and thus replicates work that has, previously, predominantly been conducted with Western samples. To begin with, disgust sensitivity was associated with moral judgments across all of the moral domains applied in the present study. However, disgust sensitivity was found to be more closely related to the endorsement of sanctity (*r* = 0.26) than to the endorsement of any other moral foundation (r: 0.11–0.19). This is consistent with Wagemans et al. (2018), whose study found that disgust sensitivity primarily to be associated with the moral concerns of purity/sanctity. The observed pattern that disgust sensitivity is strongly correlated with sanctity is not surprising as some items about endorsement of sanctity mention disgust (e.g., “Whether or not someone did something disgusting”). In addition, in the current research sanctity emerged as the strongest predicting foundation for attitudes toward homosexuality (including ATL and ATG), which is consistent with Koleva et al.’s (2012) results, where sanctity was seen to be the strongest predicting foundation for same-sex relationships. However, the current results differ from those of certain studies, such as that of Barnett et al. (2018), which may be due to a difference in the measures employed. Barnett et al. (2018) selected nine items from the Modern Homophobi Scale (MHS; Raja and Stokes, 1998) rather than the full scale in study 1 and used the Homophobia Scale (HS; Wright et al., 1999) in study 2 to assess homophobia. While the ATLG (Herek, 1998; Yu et al., 2010) in the present study selected items pertaining to cognition, emotion and behavior, and was adjusted to the Chinese context. Kiss et al. (2018) suggested that negative attitudes toward gay men may be derived from four widely held beliefs: (1) gay men’s engagement in anal intercourse is filthy; (2) gay men have been linked to AIDS; (3) gay men threaten traditional sexual morality; and (4) gay men demonstrate lack of purity on the basis of sacred scripture. The results of models B and C indicate that the endorsement of sanctity may help explain the association between disgust and negative attitude toward homosexuality, whether pertaining to gay men or lesbians. Chinese populations’ prejudice toward homosexuals is arguably unlikely to be due to religious beliefs, given that the vast majority of Chinese people are not religious. The other three beliefs outlined above by Kiss et al. (2018) could thus explain why some individuals consider gay men to be disgusting and immoral. As for negative attitudes toward lesbians, traditional sexual morality seems to be a reasonable explanation, as lesbians are not generally related to “sodomy” or AIDS.

The current study has some limitations. To begin with, this research used a sample of college students aged 30 or younger, who tend to have more liberal attitudes than the older population. The findings of the descriptive calculations demonstrated that the participants scored 28.2 on ATLG, which ranged from 0 to 80. In light of this, future research could use a more representative sample in order to increase generalizability. In addition, a self-report scale, ATLG, was used to measure explicit attitudes toward homosexuality in this research. Given that explicit attitudes toward gay men and lesbians are usually more positive than implicit attitudes (Breen and Karpinski, 2013), future research should examine the mediating role of moral foundations on the relationship between disgust and implicit attitudes toward homosexuals, or other groups that threaten traditional sex-related morality. Furthermore, this is a cross-sectional survey study, and there exist several problems with cross-sectional mediation (Cole and Maxwell, 2003; Maxwell and Cole, 2007). Namely, it is not possible to know whether the mediation model proposed in this paper actually unfolds in the predicted way over time (i.e., that disgust sensitivity predicts an endorsement of sanctity, which then predicts attitudes toward homosexual); other (bi-)directional relationships and causal mechanisms are also possible. The last, some of the Cronbach’s alphas are lower than ideal (e.g., on the subscales of the MFQ30), which has implications for the reliability of the current findings.

## Ethics Statement

This study was approved by the board of ethics at the Department of General Education, Xiamen University Tan Kah Kee College.

## Author Contributions

RW and PH conceptualized and designed the manuscript. RW, QY, PH, LS, and YG collected, analyzed, and interpreted the data and critically revised the manuscript. RW drafted the manuscript.

## Conflict of Interest Statement

The authors declare that the research was conducted in the absence of any commercial or financial relationships that could be construed as a potential conflict of interest.

## Footnotes

1 www.moralfoundations.org
